# Right Atrial-to-Right Ventricular Area: Prognostic Value in Patients With Secondary Tricuspid Regurgitation and Heart Failure

**DOI:** 10.1016/j.jacadv.2025.102334

**Published:** 2025-11-12

**Authors:** Charlotte Jantsch, Sophia Koschatko, TingTing Wu, Christoph Torrefranca, Kseniya Halavina, Varius Dannenberg, Caglayan Demirel, Christian Nitsche, Laurenz Hauptmann, Maximilian Autherith, Georg Goliasch, Christian Hengstenberg, Noemi Pavo, Philipp E. Bartko, Gregor Heitzinger

**Affiliations:** Department of Internal Medicine II, Medical University of Vienna, Vienna, Austria

**Keywords:** functional tricuspid regurgitation, heart failure, risk stratification, secondary tricuspid regurgitation

## Abstract

**Background:**

Severe secondary tricuspid regurgitation (STR) in heart failure patients is associated with excess mortality. Quantification associated risk assessment, remains challenging, specifically with contextual anatomic variability. Recent observations identified different morphological STR substrates based on predominance of ventricular or atrial remodeling, the extent of which can be expressed as right atrial/right ventricular area (RA/RV) ratio.

**Objectives:**

This study aims to investigate prognostic implications of RA/RV ratio in patients with STR.

**Methods:**

This observational study included 13,174 STR patients, 1,219 had severe STR. Patients were allocated to predominant atrial remodeling (RA/RV ratio ≥1.17) vs predominant ventricular remodeling (RA/RV ratio <1.17). The primary endpoint was all-cause mortality.

**Results:**

Both groups showed similar STR severity (effective regurgitant orifice area: 0.45 vs 0.46; *P* > 0.904, vena contracta: 13.7 vs 13.5; *P* = 0.322) and neurohumoral activation (N-terminal pro-brain natriuretic peptide 3,452 vs 3,567 *P* = 0.6). Long-term mortality was higher for predominant atrial remodeling than predominant ventricular remodeling (47% vs 39%, respectively). Spline analysis revealed increasing hazard with larger RA/RV ratio, and incline of hazard subsequently flattens in predominant atrial remodeling. Both STR remodeling types had impaired survival compared to patients at risk for severe STR (predominant atrial remodeling: HR: 2.1; 95% CI: 1.9-2.4; *P* < 0.001; predominant ventricular remodeling: HR: 1.6; 95% CI: 1.4-1.8; *P* < 0.001). This effect remained after multivariable adjustment.

**Conclusions:**

RA/RV ratio, an easily obtainable metric, associates with morphological features of valve apparatus distortion secondary to differential remodeling. Despite comparable quantified STR and neurohumoral activation, association between RA/RV ratio and mortality is robust. Whether RA/RV ratio might help identify patients benefiting from STR-tailored treatment, needs to be demonstrated by future research.

Secondary tricuspid regurgitation (STR) in heart failure (HF) patients results from maladaptive right-ventricular and right-atrial remodeling. STR is common and associated with impaired quality of life, frequent HF hospitalizations, and poor survival.^1^, ^2^, ^3^ Recent advances with transcatheter-based treatment aiming to reduce STR and improve poor clinical outcomes have moved to the center of scientific and clinical attention.^4^, ^5^, ^6^ Simultaneously, the underlying differential remodeling of the right atrium and right ventricle has been recognized to be of crucial importance for the choice of the optimal therapeutic pathway. Although clinical implications and excess mortality of STR have been well established,^1^^,^^3^^,^^7^ there has been no unified definition for further differentiation of STR. Due to heterogenicity resulting from different causes, mechanisms, and disease stages the remodeling pattern of the right heart needs to be perceived as a spectrum on which isolated secondary atrial and ventricular STR present the polar ends. Although many patients might present with overlapping features, for most either atrial or ventricular remodeling is predominant. Previous investigations proposed the use of elaborate echocardiographic markers and clinical attributes to define morphologic patterns, but thereby lacking simplicity and applicability in clinical practice.^8^, ^9^, ^10^ In this study, we sought to describe the remodeling patterns by the ratio of the right atrial/right ventricular (RA/RV) area across the HF spectrum, relate atrial and ventricular remodeling predominance to features of valve distortion, and investigate the long-term outcome related to the spectrum of RA/RV remodeling patterns.^3^^,^^11^

## Methods

### Study design

In this observational, retrospective study patients diagnosed with tricuspid regurgitation and HF from the Medical University of Vienna’s echocardiography database and longitudinal health records between 2010 and 2020 were enrolled. In adherence to current guidelines, all HF subtypes were considered and defined using left ventricular ejection fraction (LVEF) ≤40% (heart failure with reduced ejection fraction [HFrEF]), LVEF 41% to 49% (heart failure with mildly reduced ejection fraction [HFmrEF]) or LVEF ≥50%. Necessary information such as clinical signs of HF and evidence of cardiac structural or functional abnormalities were incorporated to ensure accurate diagnosis. This included diastolic dysfunction or elevated N-terminal pro-B-type natriuretic peptide >125 pg/mL levels.^12^

Patients with STR were identified and echocardiograms were re-read using a prespecified protocol. Patients with primary tricuspid valve disease, prior tricuspid valve interventions, history of endocarditis, congenital heart disease as well as insufficient echocardiographic image quality were excluded. In addition, any other patients with evidence of other significant primary valve disease were omitted from further analysis. The remaining patients were allocated to 2 groups: patients with mild or moderate STR, at risk of developing severe STR (n = 11,955) and patients with severe STR (n = 1,219). For further investigation the ratio between the RA area and the RV area was calculated using the largest areas during the cardiac cycle—that is, end-systolic phase for RA area and end-diastolic phase for the RV area. Clinical characteristics and relevant comorbidities were collected using the hospital’s electronic health records. Comorbidities were classified according to the International Statistical Classification of Diseases and Related Health Problems. A specialized research and documentation tool software developed by the Medical University of Vienna was used for data management. Laboratory parameters were analyzed from venous blood samples according to the local laboratory’s standard protocols.

The primary endpoint of this study was defined as all-cause mortality. By Austrian law, each deceased Austrian citizen must be recorded in the Austrian Death Registry, allowing for nearly complete follow-up. Ethics approval for this study was granted by the Medical University Vienna’s ethics review board (EK no. 2137).

### Echocardiographic analysis

Echocardiograms were performed using standardized transthoracic views in adherence to European guidelines^13^^,^^14^ and with commercially available ultrasound machines (Vivid E7 and E9, GE Healthcare and Acurson S2000; Siemens). Echocardiograms were re-read to acquire additional, right heart–specific morphological and functional parameters. Additional echocardiographic parameters, required for this study, were measured by 2 cardiologists, blinded to patient's status. This analysis comprised assessment of cardiac dimensions (left ventricular and RV end-diastolic diameter, RV end-diastolic and end-systolic area, end-systolic RA area, and end-systolic left atrial area and volume) and function (LVEF, tricuspid annular plane systolic excursion [TAPSE], pulsed wave Doppler S’ wave using tissue Doppler, and fractional area change), measured in apical 4-chamber-views. Tricuspid annulus was assessed mid-systole. Tenting height, tenting area, anterior and septal tricuspid leaflet angles, and coaptation defect size were also measured mid-systole, from apical 4-chamber views. Strain analysis, derived by speckle tracking echocardiography, was used to analyze right myocardial strain using the EchoPac software.^15^ Strain was acquired from 4-chamber view and obtained as RV free wall strain. As markers of RV function, TAPSE, derived from M-mode tricuspid annular excursion, and RV tissue doppler imaging measured in apical 4-chamber view were used.^15^ LVEF was calculated by the biplane Simpson method. Tricuspid regurgitation maximum velocity and velocity time integral were analyzed from continuous wave Doppler. Estimated systolic pulmonary artery pressure was derived from tricuspid regurgitation maximum velocity and approximation of RA pressure. Systematic analysis of severity of tricuspid regurgitation was conducted by assessment of tricuspid valve morphology and color Doppler in 4-chamber view. Vena contracta width and proximal isovelocity surface area radius were measured as recommended.^13^^,^^16^ Effective regurgitant orifice area (EROA) and regurgitant volume were later calculated from derived values.^7^^,^^11^ These parameters were used to grade tricuspid regurgitation as mild, moderate, or severe as recommended. To differentiate between predominantly atrial or ventricular remodeling the ratio of RA area to RV area was calculated for each patient. The median value of was used as a threshold point for separation into 2 groups.

### Statistical analysis

Continuous data, were tested for normality, are presented as median values and IQR and analyzed using Wilcoxon rank-sum test. Categorical data were analyzed using Pearson chi square test and displayed as counts and percentages. Spline analysis using restricted cubic splines was conducted to model the effect of RA to RV area ratio on all-cause mortality. Akaike information criterion was used to determine usage of 4 knots and placed as recommended. Cox proportional hazards regression analysis was used to investigate the survival of patients and theeffect size was estimated by HRs. The cox-proportional hazard assumption was assessed using log-log plots and was satisfied for each model. Both survival differences of severe STR (comparison of atrial and ventricular remodeling STR) to patients at risk of severe STR were analyzed. Patients who received tricuspid valve intervention in the follow-up period were censored in survival analyses. HRs are presented as crude hazards but also as multivariate models to adjust for possible confounding factors. A model consisting of clinical confounders with variables that likely influence survival (age, sex, coronary artery disease, serum creatinine, and RV end-diastolic diameter) was created. In a second step a bootstrap resampling derived model (body mass index, diabetes, chronic obstructive pulmonary disease, peripheral vascular disease, ischemic heart disease, left ventricular function, blood urea nitrogen, bilirubin, albumin, and γ-glutamyl transferase) was used. The latter was developed using a stepwise bootstrap resampling procedure to determine the identity the most relevant predictors. A *P* value of <0.05 was used for selection and parameters selected in over 95% of 500 performed repeats were included in the final bootstrap model. Lastly, an echocardiographic confounder model including RV end-diastolic diameter, RV end-systolic area, RV fractional area change, RV free wall strain, TAPSE, systolic pulmonary artery pressure, and presence of cardiac implantable electronic device leads in the RV, was also conducted for patients with severe STR. Survival analysis was performed in all patients and relevant remodeling-based STR subgroups and visualized using Kaplan-Meier curves. This analysis was repeated dividing the RA/RV ratio into tertials and quartiles, survival estimates can be found in Supplemental Figures 1 and 2. Further in-depth analysis was performed to assess the impact of the respective predominant right chamber remodeling STR on outcome in specific HF subgroups. Statistical analysis was computed using R-4.4.1.

## Results

### Baseline characteristics of STR predominant remodeling type

In the observational period, a total of 13,174 were diagnosed with HF and STR, of which 11,955 patients were identified to be at a risk of severe STR. Severe STR was present in 1,219 patients after the application of exclusion criteria. Patients with severe STR were stratified by the predominant atrial or ventricular remodeling, at a median RA/RV ratio of 1.17. Patients with RA/RV ratios ≥1.17 had STR with predominantly atrial remodeling (n = 609) whereas ratios <1.17 were classified as STR patients with predominantly ventricular remodeling (n = 610) (Central Illustration).

**Central Illustration:**
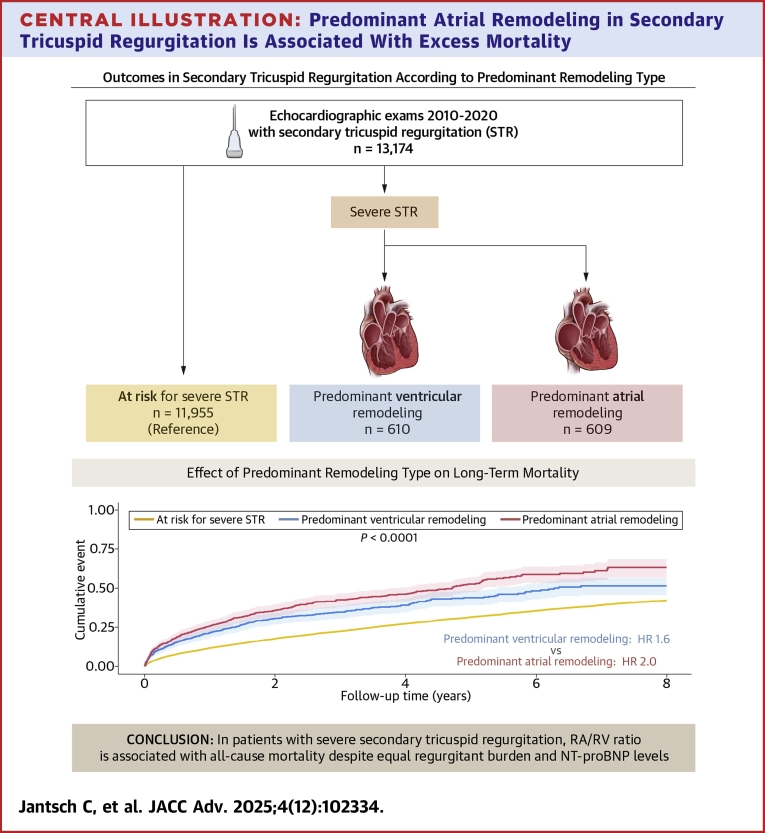
**Predominant Atrial Remodeling in Secondary Tricuspid Regurgitation Is Associated With Excess Mortality** Patients with predominant atrial remodeling have excess mortality compared to those with predominant ventricular remodeling in the setting of severe secondary tricuspid regurgitation and heart failure. A/RV ratio = right atrial-to-right venticular area ratio; NT-proBNP = N-terminal pro-B-type natriuretic peptide.

A matched sample cohort (n = 100) at risk of severe STR had RA/RV ratio of 0.93, further details on normal cardiac dimensions may be found in Supplemental Table 1. Patients with STR and atrial remodeling were older than those with ventricular remodeling (77 [IQR 70-82] vs 71 [IQR 61-79], *P* < 0.001). Overall distribution of HF subtypes was similar in both remodeling types (*P* = 0.137). HF with preserved ejection fraction was most frequently diagnosed in both atrial and ventricular predominant remodeling types, at 46% each. Atrial fibrillation occurred more often in STR with predominant atrial remodeling than predominant ventricular remodeling (58% vs 39%, *P* < 0.001), whereas other comorbidities were similar between both groups (Table 1). Severe concomitant secondary mitral regurgitation was diagnosed in 39% (n = 235) of patients with atrial remodeling as compared to 32% (n = 189) in ventricular remodeling (*P* < 0.001). Both STR remodeling types presented with similarly elevated N-terminal pro-B-type natriuretic peptide levels (predominant atrial remodeling: 3,452 [1,600-7,755] pg/mL; predominant ventricular remodeling: 3,567 [1,718-8,155] pg/mL, *P* = 0.598).

**Table 1:** Baseline Characteristics

Parameter	At Risk for Severe STR (n = 11,955)	STR With Predominant Ventricular Remodeling (n = 610)	STR With Predominant Atrial Remodeling (n = 609)	*P* Value^a^
Clinical characteristics				
Age, y	70 (61-77)	71 (61-79)	77 (70-82)	**<0.001**
Female	3,911 (33%)	230 (38%)	295 (48%)	**<0.001**
Body mass index	27.6 (24.6-31.2)	27.2 (24.0-31.9)	25.6 (23.3-29.0)	**<0.001**
Comorbidities				
Hypertension	7,423 (63%)	296 (49%)	312 (52%)	0.40
Hyperlipidemia	4,162 (35%)	147 (25%)	138 (23%)	0.50
Diabetes type II	3,146 (27%)	142 (24%)	106 (18%)	**0.009**
Coronary artery disease	6,019 (50%)	246 (40%)	237 (39%)	0.60
Atrial fibrillation	3,370 (29%)	231 (39%)	349 (58%)	**<0.001**
Cerebral artery disease	2,155 (23%)	83 (17%)	92 (18%)	0.50
Peripheral artery disease	2,870 (25%)	120 (20%)	134 (22%)	0.40
Chronic obstructive pulmonary disease	1,547 (13%)	88 (15%)	99 (17%)	0.40
Laboratory measurements				
NT-proBNP, pg/mL	1,041 (383-2,916)	3,567 (1,718-8,155)	3,452 (1,600-7,755)	0.60
Red blood cell count, T/L	4.3 (3.8-4.8)	4.2 (3.7-4.8)	4.2 (3.8-4.7)	0.60
Hemoglobin, g/dL	12.8 (11.1-14.2)	12.1 (10.6-13.9)	12.5 (11.0-13.6)	0.20
Platelets, g/L	221 (178-275)	213 (169-265)	207 (170-259)	0.90
White blood cell count, g/L	7.5 (6.1-9.4)	7.5 (6.1-9.2)	7.2 (5.9-8.7)	**0.015**
Creatinin, mg/dL	1.0 (0.9-1.3)	1.2 (0.9-1.6)	1.1 (0.9-1.4)	**0.008**
Blood urea nitrogen, mg/dL	18 (14-25)	23 (16-36)	22 (16-31)	**0.034**
Bilirubin, mg/dL	0.6 (0.4-0.8)	0.8 (0.5-1.3)	0.8 (0.6-1.2)	0.90
Albumin, g/L	39.0 (34.6-42.3)	37.7 (33.4-41.2)	37.8 (33.6-41.4)	0.60
a-Amylase, U/L	54 (39-74)	53 (37-74)	50 (37-74)	0.50
Cholinesterase, kU/L	6.5 (5.0-7.9)	5.0 (3.9-6.5)	5.2 (4.0-6.6)	0.30
Alkaline phosphatase, U/L	72 (58-92)	85 (66-118)	84 (64-110)	0.20
Aspartate transaminase, U/L	26 (20-36)	28 (22-38)	28 (22-38)	0.60
Alanine transaminase, U/L	24 (17-37)	24 (17-37)	23 (16-35)	0.50
Gamma glutamyl transferase, U/L	38 (22-74)	76 (40-151)	66 (34-125)	**0.006**
Lactat dehydrogenase, U/L	210 (175-267)	231 (191-285)	231 (195-280)	0.80
Creatine kinase, U/L	88 (54-147)	74 (46-121)	72 (48-121)	>0.90
HbA1c, %	5.9 (5.5-6.5)	6.0 (5.6-6.6)	5.9 (5.6-6.3)	0.13
Cholesterol, mg/dL	162 (131-196)	136 (109-166)	142 (119-171)	**0.003**
C-reactive protein, mg/dL	0.7 (0.2-2.6)	1.0 (0.4-2.8)	0.9 (0.3-2.5)	0.20
Heart failure subgroup				0.14
HFpEF	7,021 (59%)	279 (46%)	282 (46%)	
HFmrEF	2,859 (24%)	107 (18%)	130 (21%)	
HFrEF	2,075 (17%)	224 (37%)	197 (32%)	
Secondary mitral regurgitation				**<0.001**
Mild	3,728 (32%)	77 (13%)	26 (4.4%)	
Moderate	6,931 (60%)	322 (55%)	334 (56%)	
Severe	819 (7.1%)	189 (32%)	235 (39%)	
Tricuspid valve intervention^b^	46 (0.4%)	18 (3.0%)	31 (5.1%)	**0.002**

Values are median (IQR) or n (%). **Bold***P*-values are statistically significant.

HbA1c = glycated hemoglobin; HFmrEF = heart failure with mildly reduced ejection fraction, HFpEF = heart failure with preserved ejection fraction; HFrEF = heart failure with reduced ejection fraction; NT-proBNP = N-terminal pro-brain natriuretic peptide; STR = secondary tricuspid regurgitation.

a Wilcoxon rank-sum test; Pearson chi-square test. *P* values calculated between predominant atrial and ventricular remodeling secondary tricuspid regurgitation.

b Tricuspid valve intervention (either surgical or transcatheter) in the follow-up period.

### Echocardiographic characterization of STR phenotype

Detailed echocardiographic analysis of tricuspid valve geometry, cardiac dimension and function, and markers of regurgitation severity are presented in Table 2. Patients with predominantly atrial remodeling had significantly less tenting area (predominant atrial remodeling: 100 [49-162] mm^2^, predominant ventricular remodeling: 135 [85-190] mm^2^, *P* < 0.001), larger RA area (predominant atrial remodeling: 30 [26-37] cm^2^, predominant ventricular remodeling: 26 cm^2^ [21-31] cm^2^, *P* < 0.001) and smaller end-diastolic RV area (predominant atrial remodeling: 21 [17-25] cm^2^, predominant ventricular remodeling 27 [22-33] cm^2^, *P* < 0.001). RV function, as expressed by RV fractional area change and RV free wall strain was better in STR with predominant atrial remodeling (*P* < 0.001), whereas ejection fraction did not differ between the 2 groups (*P* = 0.874). Markers of tricuspid regurgitation severity were similar in both STR subgroups (Table 2); however, STR with ventricular remodeling presented with higher regurgitant volume (predominant atrial remodeling: 45 [31-64] mL, predominant ventricular remodeling 50 [35-71] mL, *P* = 0.004).

**Table 2:** Echocardiographic and Detailed Morphological Characteristics According to STR With Predominant Atrial or Ventricular Remodeling

Parameter	STR With Predominant Ventricular Remodeling (n = 610)	STR With Predominant Atrial Remodeling (n = 609)	*P* Value^a^
Tricuspid valve dimensions			
Tricuspid annulus, mm	34 (30-38)	34 (30-39)	0.473
Tenting height, mm	7.8 (5.7-10.1)	6.2 (4.0-8.7)	**<0.001**
Tenting area, mm^2^	135 (85-190)	100 (49-162)	**<0.001**
Anterior leaflet angle	25 (19-30)	21 (14-27)	**<0.001**
Septal leaflet angle	29 (23-36)	25 (18-32)	**<0.001**
Coaptation defect, mm	0.5 (0.5-0.5)	0.5 (0.5-0.5)	0.362
Cardiac dimensions and function			
RA/RV ratio	0.96 (0.84-1.06)	1.42 (1.28-1.65)	**<0.001**
Left atrial volume, mL	92 (66-121)	110 (82-144)	**<0.001**
Left atrial area, cm^2^	28 (23-33)	31 (26-37)	**<0.001**
Right atrial area, cm^2^	26 (21-31)	30 (26-37)	**<0.001**
Left ventricular end systolic diameter, mm	39 (32-48)	38 (32-46)	**0.005**
Left ventricular ejection fraction (LVEF), %	44 (31-55)	43 (32-53)	0.874
Right ventricular end-systolic area, cm^2^	17 (13-23)	13 (10-16)	**<0.001**
Right ventricular end-diastolic area, cm^2^	27 (22-33)	21 (17-25)	**<0.001**
Right ventricular fractional area change, %	36 (27-44)	40 (30-48)	**<0.001**
Right ventricular free wall strain, %	−15 (−20 to −10)	−17 (−22 to −12)	**<0.001**
TAPSE, mm	15.0 (12.0-18.0)	15.5 (13.0-18.0)	0.536
Right ventricle tissue Doppler imaging m/s	0.09 (0.07-0.12)	0.10 (0.08-0.12)	0.675
Tricuspid regurgitation			
Tricuspid regurgitation velocity m/s	3.3 (3.0-3.8)	3.2 (2.8-3.5)	**<0.001**
Tricuspid regurgitation velocity time integral cm	108 (91-129)	99 (86-114)	**<0.001**
Proximal isovelocity surface area, cm	1.0 (0.8-1.2)	1.0 (0.8-1.1)	0.065
Vena contracta width, mm	13.5 (10.0-16.8)	13.7 (10.2-17.1)	0.322
EROA, cm^2^	0.5 (0.3-0.7)	0.5 (0.3-0.7)	0.904
Regurgitant volume, ml	50 (35-71)	45 (31-64)	**0.004**
Inferior vena cava, mm	24.0 (21.0-26.6)	24.0 (21.0-27.0)	0.582
Pulmonary artery pressure, mm Hg	56 (45-69)	52 (43-61)	**<0.001**

Values are Median (IQR). **Bold***P*-values are statistically significant.

EROA = effective regurgitant orifice area; RA/RV ratio = right atrial-to-right ventricular area ratio; TAPSE = tricuspid annular plane systolic excursion; other abbreviations as in Table 1.

a Wilcoxon rank sum test; Pearson’s Chi-squared test.

### Long-term outcome of STR by RA/RV ratio

**Figure 1:**
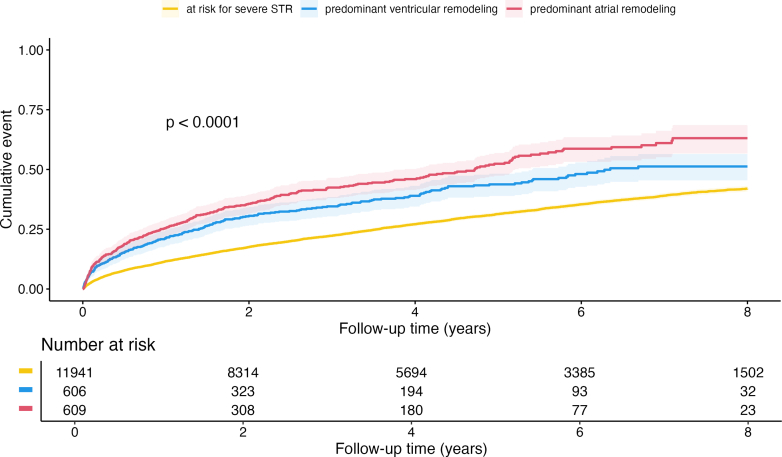
**Mortality of Secondary Tricuspid Regurgitation** Long-term survival, after 8 years, of patients with secondary tricuspid regurgitation, log-rank *P* > 0,001. Yellow: at risk for STR, blue: STR with predominant ventricular remodeling, red: STR with predominant atrial remodeling. STR = secondary tricuspid regurgitation.

**Figure 2:**
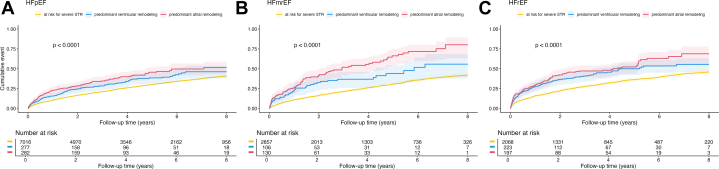
**Survival of Secondary Tricuspid Regurgitation Across the Heart Failure Spectrum** Each panel represents Kaplan-Meier survival analysis of patients with STR and heart failure by STR remodeling type in a heart failure subgroup (A) in patients with HFpEF (log-rank *P* > 0.001), (B) in HFmrEF (log-rank *P* > 0.001), and (C) in HFrEF (log-rank *P* > 0.001). Yellow: at risk for STR, blue: STR with predominant ventricular remodeling, red: STR with predominant atrial remodeling. HFmrEF = heart failure with mildly reduced ejection fraction; HFpEF = heart failure with preserved ejection fraction; HFrEF = heart failure with reduced ejection fraction; other abbreviations as in Figure 1.

At 4 years, STR with predominantly atrial remodeling had the highest fatal event rate compared to STR with ventricular remodeling and patients at-risk for STR (predominant atrial remodeling: 58% [n = 244], predominant ventricular remodeling: 50% [n = 204], at risk for STR: 46% [2,850], *P* < 0.001). This trend of higher all-cause mortality in STR with atrial remodeling was also reflected at later follow-up times (Figure 1). With patients at risk of severe STR as reference, both STR with atrial and ventricular phenotype had significantly increased mortality in unadjusted cox regression analysis, (HR predominant atrial remodeling: 2.1 [1.8-2.1], *P* < 0.001, predominant ventricular remodeling: 1.6 [1.4-1.8], *P* < 0.001). Directly comparing the predominant atrial remodeling and predominant ventricular remodeling groups showed increased hazard of the predominant atrial remodeling type, (HR predominant atrial remodeling: 1.3 [1.1-1.50], *P* = 0.003). The adverse effect of predominant atrial and predominant ventricular remodeling remained evident even after adjustment using a bootstrap confounder and a clinical confounder model (Table 3, extended version Supplemental Table 2). The third, multivariable echocardiographic confounder model showed increased hazard for predominant atrial remodeling (HR: 1.6 [1.2-2.0]; *P* < 0.001) with predominant ventricular remodeling as reference (Supplemental Table 3). Further differentiation revealed a similar adverse impact of STR with atrial remodeling independent of the underlying HF subtypes (Figures 2A, 2B, and 2C, Supplemental Table 4). Consistent with the overall risk-profile, mortality was highest with predominant atrial remodeling in combination with HFrEF.

**Table 3:** Cox Regression Models Assessing Impact of Predominant STR Remodeling Patterns on Long-Term Mortality

STR Group	Univariate Model		Multivariable Bootstrap Model^a^		Multivariable Clinical Confounder Model^b^	
	HR (95% CI)	*P* Value	HR (95% CI)	*P* Value	HR (95% CI)	*P* Value
At risk severe STR	Reference		Reference		Reference	
Severe STR with predominant ventricular remodeling	1.6 (1.4-1.8)	**<0.001**	1.3 (1.1-1.5)	**0.002**	1.2 (1.1-1.4)	**0.009**
Severe STR with predominant atrial remodeling	2.1 (1.9-2.4)	**<0.001**	1.8 (1.6-2.1)	**<0.001**	1.4 (1.2-1.6)	**<0.001**

**Bold***P*-values are statistically significant.

Abbreviations as in Table 1.

a The multivariate bootstrap model includes the following parameters: body mass index, diabetes mellitus, chronic obstructive pulmonary disease, peripheral artery disease, coronary artery disease, blood urea nitrogen, total bilirubin, albumin, gamma-glutamyltransferase, and left ventricular ejection fraction.

b The clinical multivariate cox regression model is composed of age, sex, right ventricular end-diastolic diameter, coronary artery disease and serum creatinine. Results are shown as HR (95% CI).

**Figure 3:**
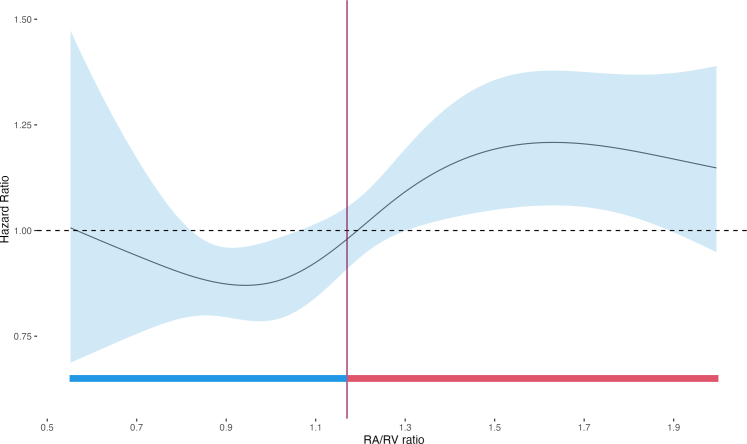
**Spline Curves of Right Atrial-to-Right Venticular Area Ratio** Restricted cubic spline model of the right atrial to ventricular area ratio and hazard ratio for mortality. The red line represents the cut-off of 1.17, used to differentiate between predominant ventricular remodeling (<1.17) predominant atrial remodeling (>1.17). RA/RV ratio = right atrial-to-right venticular area ratio.

Spline curve analysis, depicted in Figure 3, illustrates the association of the RA/RV ratio to mortality. Patients with higher ratios, representing more advanced atrial remodeling, had increased risk of mortality. Subgroup analysis was conducted for both remodeling types (Supplemental Figures 3, 4). The adverse impact of severe STR was evident in most subgroups, except patients with predominant ventricular remodeling and severely reduced RV function or severe RV dilatation. All-cause mortality was markedly higher in younger STR patients (p-for-interaction = 0.035) with predominant atrial remodeling. The association between STR and all-cause mortality was more pronounced for patients with ventricular remodeling and concomitant ischemic heart disease (*P* for interaction = 0.002).

## Discussion

This large-scale observational study investigates the impact of atrial and ventricular remodeling in severe STR using the RA/RV ratio. This study provides a comprehensive echocardiographic characterization of remodeling phenotypes and investigates the prognostic significance of the RA/RV ratio, on long-term survival. The main findings are as follows.

1. Both severe STR with atrial and ventricular remodeling are at significantly increased risk of mortality in comparison to patients at risk of severe STR.
2. STR with predominant atrial remodeling exhibits the highest mortality, even after adjusting for confounders and irrespective of the underlying HF subtype. This is specifically remarkable as neurohumoral activation as well as quantified tricuspid regurgitation severity were comparable in both phenotypes.
3. The RA/RV ratio delineates a spectrum of morphologic remodeling patterns associated with an increasing risk relationship among patients with severe STR. Furthermore, it reflects profound morphological diversity among the pathophysiological remodeling spectrum ranging from pronounced atrial enlargement with minimal leaflet distortion to severe ventricular remodeling accompanied by profound geometric distortion of the valve apparatus.

### Heterogenous concepts of atrial and ventricular STR and relation to predominant remodeling patterns

STR gained increasing recognition as an important clinical entity due to high prevalence, severe impact on symptomatic status and quality of life as well as associated mortality.^1^^,^^3^^,^^17^ Transcatheter treatment options have expanded the number of patients for whom valve repair or replacement can be considered;^18^^,^^19^ nevertheless, there remains uncertainty about the impact of these treatment strategies beyond symptom and quality of life improvement. This indicates an unmet need for improved phenotypic characterization and the associated impact on outcome to generate hypotheses toward optimized patient selection for valve repair and replacement therapies.

Recognition of the complex pathophysiology of STR has led to the emergence of multiple, heterogenous definitions of STR phenotypes.^8^^,^^10^^,^^11^ Thus far, STR classification in part has been based on the presence of clinical comorbidities, resulting in the notion that presence of atrial fibrillation is a necessity for atrial STR.^8^ A recent study defined ventricular STR in the presence of left-sided cardiac disease, pulmonary hypertension or RV dysfunction, and atrial STR as coinciding atrial fibrillation.^8^^,^^20^ Therefore, by definition, no patients in the ventricular STR group had atrial fibrillation.^9^ Echocardiographic aspects, such as tenting height, LVEF, and RV dimensions, have, however, also been used to define atrial STR.^10^ This uncertainty is reflective of the complex and heterogenous remodeling patterns in STR. The use of the RA/RV ratio is not intended to strictly differentiate atrial from ventricular STR but rather to characterize the remodeling spectrum and phenotypes. It represents one of several metrics that could complementarily contribute to a more comprehensive understanding of the heterogenous changes and their role in delineating predominating remodeling in STR. This paper investigates an intuitive and applicable approach by using the RA/RV ratio, where atrial and ventricular STR exist as opposite ends.

### Morphologic aspects of STR remodeling types

In this paper we examine morphologic and secondary differences between predominant remodeling patterns and distinguish patients based on right-sided morphology. Patients with larger RV area in comparison to the RA area were classified as STR with predominantly ventricular remodeling. These patients had worse RV function, determined by fractional area change and RV free wall strain (Table 2). Tricuspid valve morphology also differs, with significantly larger tenting height and area in these patients. This suggests the underlying mechanism behind predominant ventricular remodeling to be primarily due to leaflet tethering or restriction.^11^ STR patients with predominantly atrial remodeling had flatter tricuspid annuli and less leaflet tenting, indicating mechanisms of tricuspid regurgitation likely involve atrial and annular maladaptation.^9^^,^^11^ This concept is reinforced by the larger RA dimensions. Although the morphologic aspects of the tricuspid valve and the functional echocardiographic parameters are divergent, the severity of tricuspid regurgitations is comparable among both analyzed groups. In addition, neurohumoral activation was similar, nevertheless patients with predominant atrial remodeling exhibited excess mortality.

Although atrial fibrillation and annular dilation play an important role in atrial STR, its development is a multifactorial process, and may occur without presence of atrial fibrillation.^9^ Atrial enlargement and comparative ratio to the RV area should be taken into consideration, to best establish leading cause of STR. An observational study in South Korea, conducting analysis of STR progression in the presence of atrial fibrillation demonstrated that large RA area to RV area ratio was a significant risk factor for the progression of STR.^21^ This highlights the role of atrial enlargement in progression of STR. Rapid progression of STR was also associated with higher mortality, regardless of initial severity.^22^ Progression of STR was quantified by increasing enlargement of atrial area, tricuspid annular diameter, and left ventricular diameter, not by EROA or vena contracta.^22^ Similarly, another study investigating STR persistence and progression in patients undergoing transcatheter aortic valve replacement found a larger RA/RV ratio (>1.13), an independent predictor for STR persistence after intervention, which was furthermore associated with an increased all-cause mortaility.^23^ Similar results were observed for secondary mitral regurgitation.^24^

Due to this reasoning, the ratio of RA area to RV area plays an important part in classifying ventricular and atrial phenotypes of STR and may represent an important prognostic factor for disease progression.

### Disproportionate secondary tricuspid regurgitation

Recent research has explored the concept of disproportionate secondary mitral regurgitation, in which the regurgitant lesions with similar EROA have a different impact in the context of different ventricular size and function, leading to excess mortality in those with disproportionate secondary mitral regurgitation compared to those with proportionate secondary mitral regurgitaiton.^25^, ^26^, ^27^ Although this concept has been explored for the mitral valve, only scarce research exists for application to the tricuspid valve. A proposed concept suggests a ratio of regurgitant volume to RV end-diastolic volume to determine proportionality. Similarly, patients with disproportionate STR have excess all-cause mortality compared to those with proportionate STR.^28^ In this study, patients with predominant ventricular remodeling have larger RV dimensions despite comparable quantified tricuspid regurgitation EROA, thus representing the proportionate STR group while patients with predominant atrial remodeling, hence smaller RV dimensions might indicate more disproportionate STR. Therefore, by exhibiting comparable TR severity and different degree of RV dilation, the disproportionate STR concept may deliver an explanation for worse outcomes of patients with predominant atrial remodeling.

### STR phenotypes across the heart failure spectrum

This study defines HF subgroups in adherence to current guidelines, thus ensuring accurate representation of STR remodeling types across the HF spectrum. Previous evidence has indicated patients with severe tricuspid regurgitation and HFrEF have higher mortality,^7^^,^^29^ a phenomenon which also applies to HFmrEF and heart failure with preserved ejection fraction (HFpEF) patients, as demonstrated in more recent studies.^3^^,^^30^

Formerly, it has been expected that patients with ventricular STR primarily had HFrEF whereas atrial STR patients tended to have HFpEF.^9^ This concept, however, does not apply to this patient cohort and distribution by predominant atrial or ventricular remodeling. Distribution of predominant atrial and ventricular remodeling of STR across the HF spectrum was balanced, with HFpEF being the most prevalent HF subgroup of both STR phenotypes. HFpEF was present in 45% of patients and 51% of predominant atrial remodeling patients had either HFmrEF or HFrEF. Thus, any stage of HF can occur with each type of predominant remodeling. The pervading excess mortality of large RA/RV ratio throughout the HF spectrum reiterates the importance and prognostic value of this parameter.

## Clinical implications

Severe tricuspid regurgitation is common, affecting 0.55% of the population and 4% of patients aged over 75 years.^1^ In a steadily aging population, the prevalence of STR is expected to continuously rise. Pharmacological treatment options for STR are limited and data suggest that tricuspid regurgitation does not necessarily improve with HF treatment and may even progress under optimal medical therapy.^29^

Isolated tricuspid valve surgery is associated with a high postoperative, often prohibitive mortality.^11^ Alternatively, percutaneous tricuspid repair and replacement options now offer a safe and comparably low-risk treatment option,^31^^,^^32^ with both the TRILUMINATE pivotal trial and TRISCEND study establishing good safety, effective reduction of tricuspid regurgitation, and increased quality of life.^18^^,^^19^^,^^33^ Broad availability of such devices necessitates careful patient selection, taking into consideration the right-sided cardiac morphology and imaging quality, to ensure best patient care.

The RA/RV ratio identifies STR patient subgroups with adverse long-term outcomes. Patients with predominantly atrial remodeling have an increased risk of mortality compared to patients with ventricular remodeling, despite comparable comorbidity burden, neurohumoral activation, and better RV function. In summary, these results suggest patients with predominant atrial remodeling represent a high-risk group within patients with severe STR. In addition, transcatheter ablation or cardioversion may also be a feasible treatment option for patients with STR and atrial fibrillation, as conversion to sinus rhythm has shown improvement of tricuspid regurgitation and reduction of RA size.^13^^,^^34^^,^^35^ The question remains, if this constitutes a preventive measure for patients at risk for STR. There are limited data available on the impact and choice of treatment modality in these patients. Prospective studies are needed to guide the clinician’s decision-making and provide treatment strategies tailored to the patient.

### Strengths and limitations

Strengths of this paper are the large study population from a tertiary care center and granular data allowing for accurate HF diagnosis. In addition, near complete follow-up data allow for accurate long-term assessment of patient survival over the course of 8 years. Comprehensive analysis of echocardiographic images, applying the methodology recommended in current guidelines, was conducted to analyze the tricuspid valve apparatus and regurgitation parameters. This was a retrospective data analysis and therefore results must be interpreted with caution. Specific biases, such as selection and referral bias cannot be excluded. Using a data-derived threshold for the RA/RV ratio is an additional limitation. The RA/RV ratio depicts a spectrum of phenotypes with different clinical characteristics and outcomes. Furthermore, data on HF medication and HF hospitalizations were not available and would likely provide further insights. All-cause mortality was chosen to fully reflect mortality in HF and STR; however, additional cardiovascular endpoints may provide further insights.

## Conclusions

The RA/RV ratio and resulting remodeling patterns aid in identification of patient subgroups with increased mortality in STR. In particular, patients with predominant atrial remodeling represent a STR subgroup suffering from excess mortality. By accounting for pathologic changes to the right heart and tricuspid valve apparatus causal to STR, we ascertain right-sided cardiac morphology as a deciding factor for excess mortality. This unfavorable effect on survival prevails after adjustment for clinical covariates and was evident across the HF spectrum. The RA/RV ratio is an echocardiographic parameter easily applicable in clinical practice and may be beneficial to early detection and risk-stratification in patients with STR.

## Funding support and author disclosures

The authors have reported that they have no relationships relevant to the contents of this paper to disclose.
