# Supplementary material for: Right Atrial-to-Right Ventricular Area: Prognostic Value in Patients With Secondary Tricuspid Regurgitation and Heart Failure
Source: JACC Adv. 2025 Nov 12;4(12):102334. doi: 10.1016/j.jacadv.2025.102334 (PMC12662097; doi:10.1016/j.jacadv.2025.102334)
Supplement: Supplemental Tables 1 to 4 and Supplemental Figures 1 to 4 [file mmc1.docx]

**Supplemental Appendix**

**To the manuscript entitled “Right atrial-to-right ventricular area: Prognostic value in patients with secondary tricuspid regurgitation and heart failure” by Jantsch et al.**

**Supplemental Table 1………………………………………………………………………2**

**Supplemental Table 2………………………………………………………………………3**

**Supplemental Table 3……………………………………………………………….…..…4**

**Supplemental Table 4…………………………………………………………………...…5**

**Supplemental Figure 1……………………………………………………………….…….7**

**Supplemental Figure 2………………………………………………………………..……8**

**Supplemental Figure 3……………………………………………………………..…..…..9**

**Supplemental Figure 4……………………………………………………………..….….10**

**Supplemental Table 1: RA/RV ratio in across study populations with varying STR severity**

|  | **No TR/normal cardiac dimensions** | **At risk for severe STR** | **Severe STR** | |
| --- | --- | --- | --- | --- |
| **STR severity** | no TR | mild & moderate | severe-torrential | |
| **Sample size, n** | 100 | 100 | 1219 | |
| **RA/RV ratio** | 0.70 | 0.93 | 1.17 | |
|  |  |  | **Predominant ventricular remodeling** | **Predominant atrial remodeling** |
| **Sample size, n** | - | - | 610 | 609 |
| **RA/RV ratio** | **-** | - | 0.96 | 1.42 |
|  |  |  |  |  |

*RA/RV ratio: right atrial-to-right ventricular area ratio, STR: secondary tricuspid regurgitation*

**Supplemental Table 2 (extension of manuscript Table 3): Output of multivariable cox regression model for predominant STR remodeling-type**

|  | **Multivariable bootstrap model** | | **Multivariable**  **clinical confounder model** | |
| --- | --- | --- | --- | --- |
|  | **HR** **(95% CI**) | **p-value** | **HR** **(95% CI**) | **p-value** |
| **at risk severe STR** | Reference |  | Reference |  |
| **severe STR with predominant ventricular remodeling** | 1.3 (1.1-1.5) | **0.004** | 1.2 (1.0- 1.4) | **0.012** |
| **severe STR with predominant atrial remodeling** | 1.7 (1.5- 2.0) | **<0.001** | 1.4 (1.2- 1.5) | **<0.001** |
| Body mass index | 0.97 (0.96-0.97) | **<0.001** | - | **-** |
| Diabetes type II | 1.30 (1.20-1.41) | **<0.001** | - | **-** |
| Chronic obstructive  pulmonary disease | 1.51 (1.38-1.65) | **<0.001** | - | **-** |
| Peripheral artery disease | 1.45 (1.35-1.57) | **<0.001** | - | **-** |
| Coronary artery disease | 0.83 (0.77-0.89) | **<0.001** | 1.03 (0.97-1.10) | 0.34 |
| Blood urea nitrogen, mg/dl | 1.02 (1.01-1.02) | **<0.001** | - | **-** |
| Bilirubin, mg/dl | 1.07 (1.05-1.09) | **<0.001** | - | **-** |
| Albumin, g/l | 0.94 (0.93-0.94) | **<0.001** | - | **-** |
| Gamma glutamyl transferase, U/l | 1.00 (1.00-1.00) | **<0.001** | - | **-** |
| Left ventricular function | 1.04 (1.01-1.07) | **0.004** | - | **-** |
| Age, years | - | **-** | 1.05 (1.04-1.05) | **<0.001** |
| Sex (female) | - | **-** | 0.92 (0.86-0.99) | **0.019** |
| Creatinin, mg/dl | - | **-** | 1.22 (1.19-1.24) | **<0.001** |
| Right ventricular end-diastolic diameter | **-** | **-** | 1.02 (1.02-1.03) | **<0.001** |
| Right ventricular function | **-** | **-** | 1.71 (1.44-2.05) | **<0.001** |

Cox Regression models of predominant remodeling type. Output of each variable added the multivariable bootstrap and clinical confounder models. Results are shown as hazard ratios (95% confidence interval). *CI: confidence interval, HR: hazard ratio, STR: secondary tricuspid regurgitation.*

**Supplemental Table 3: Multivariable cox regression model adjusted for echocardiographic parameters by predominant STR remodeling-type**

| **Multivariable echocardiographic confounder model** | **HR** **(95% CI**) | **p-value** |
| --- | --- | --- |
| **severe STR with predominant  ventricular remodeling** | Reference | **-** |
| **severe STR with predominant  atrial remodeling** | 1.6 (1.2- 2.0) | **<0.001** |
| Right ventricular end-diastolic diameter | 1.0 (1.0-1.0) | 0.20 |
| Right ventricular end-systolic area | 1.0 (1.0-1.0) | 0.74 |
| Right ventricular fractional area change | 1.0 (1.0-1.0) | 0.58 |
| Right ventricular free wall strain | 1.0 (1.0-1.0) | 0.24 |
| Tricuspid annular plane systolic excursion | 1.0 (0.9-1.0) | **0.006** |
| Systolic pulmonary artery pressure | 1.0 (1.0-1.0) | **0.027** |
| Cardiac implantable electronic device | 1.2 (0.9-1.6) | 0.28 |

Cox Regression models of predominant remodeling type adjusted for the following echocardiographic parameters: right ventricular end-diastolic diameter, right ventricular end-systolic area, right ventricular fractional area change, right ventricular free wall strain, tricuspid annular plane systolic excursion, systolic pulmonary artery pressure, and presence of a cardiac implantable electronic device*. STR: secondary tricuspid regurgitation.*

**Supplemental Table 4: Crude and multivariable cox regression model for predominant STR remodeling-type across the heart failure spectrum**

|  | **Univariate model** | | **Bootstrap model ^a^** | | **Clinical multivariate model ^b^** | |
| --- | --- | --- | --- | --- | --- | --- |
|  | HR^1^ (95% CI^1^) | p-value | HR^1^ (95% CI^1^) | p-value | HR^1^ (95% CI^1^) | p-value |
| **HFpEF** |  | |  | |  | |
| at risk severe STR | Reference | | Reference | | Reference | |
| severe STR with predominant ventricular remodeling | 1.4 (1.1- 1.7) | **0.003** | 1.2 (0.9- 1.5) | 0.19 | 1.1 (0.9- 1.3) | 0.55 |
| severe STR with predominant atrial remodeling | 1.7 (1.4- 2.1) | **<0.001** | 1.5 (1.2- 1.8) | **<0.001** | 1.2 (1.0- 1.5) | 0.052 |
| **HFmrEF** |  | |  | |  | |
| at risk severe STR | Reference | | Reference | | Reference | |
| severe STR with predominant ventricular remodeling | 1.8 (1.3 - 2.4) | **<0.001** | 1.5 (1.0- 2.1) | **0.038** | 1.2 (0.9- 1.7) | 0.28 |
| severe STR with predominant atrial remodeling | 2.9 (2.3- 3.7) | **<0.001** | 2.7 (2.1- 3.6) | **<0.001** | 1.7 (1.3- 2.2) | **<0.001** |
| **HFrEF** |  | |  | |  | |
| at risk severe STR | Reference | | Reference | | Reference | |
| severe STR with predominant ventricular remodeling | 1.6 (1.3-2.0) | **<0.001** | 1.3 (1.1- 1.7) | **0.019** | 1.3 (1.0- 1.6) | **0.030** |
| severe STR with predominant atrial remodeling | 2.0 (1.6- 2.4) | **<0.001** | 1.8 (1.4- 2.3) | **<0.001** | 1.3 (1.0- 1.6) | **0.045** |

Cox Regression models of predominant remodeling type across the heart failure spectrum. The multivariate bootstrap model includes body mass index, diabetes mellitus, chronic obstructive pulmonary disease, peripheral artery disease, coronary artery disease, blood urea nitrogen, total bilirubin, albumin, gamma-glutamyl-transferase, and left ventricular ejection fraction. ^a^ The clinical multivariate cox regression model is composed of age, gender, right ventricular end-diastolic diameter, coronary artery disease and serum creatinine. ^b^ Results are shown as hazard ratio (95% confidence interval).

*CI: confidence interval HFmrEF: heart failure with mildly reduced ejection fraction, HFpEF: heart failure with preserved ejection fraction, HFrEF: heart failure with reduced ejection fraction, HR: hazard ratio, STR: secondary tricuspid regurgitation.*

**Supplemental Figure 1: Long-term Kaplan-Meier survival analysis of patients with secondary tricuspid regurgitation (STR) with severe STR divided into tertials**

**
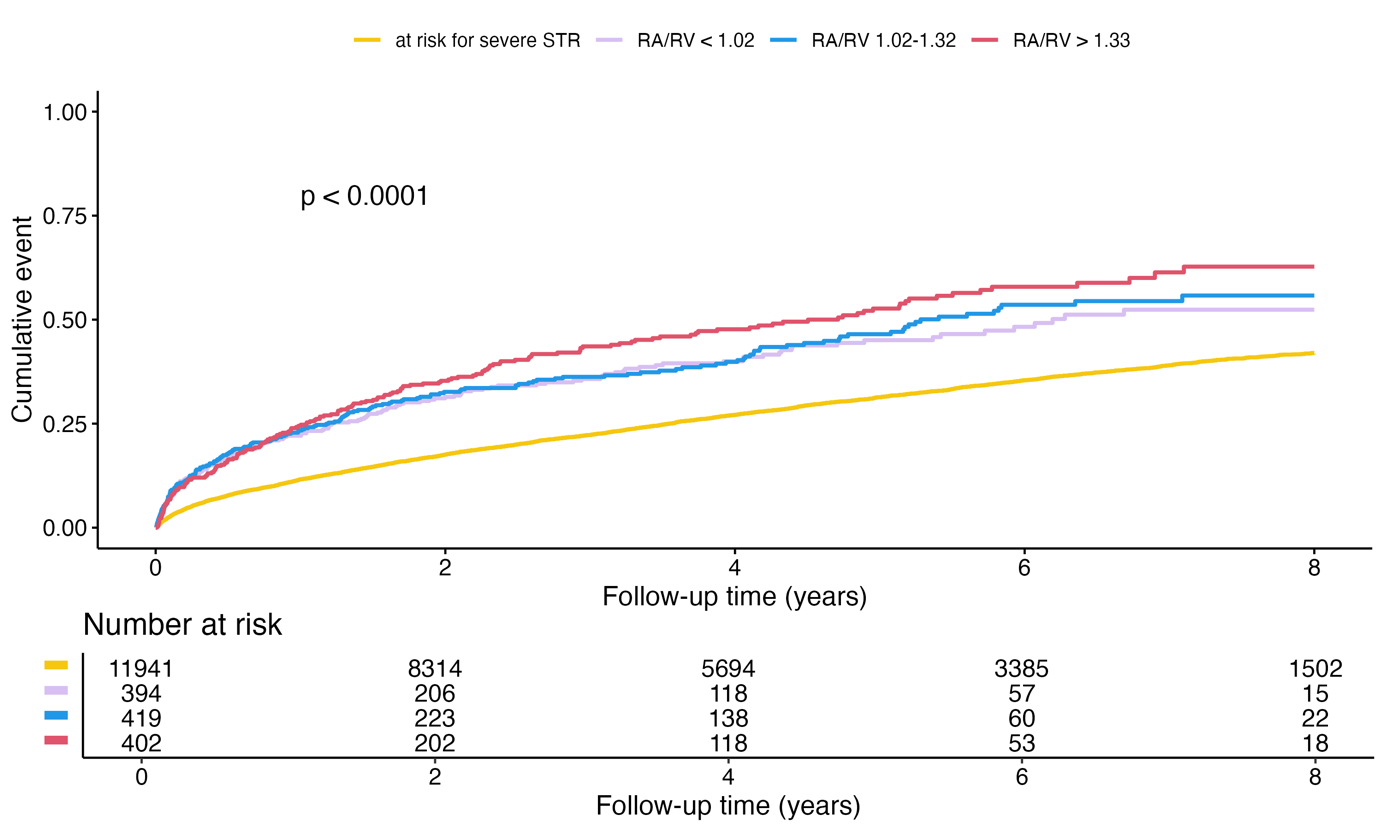
**

Log-rank p>0,001. Yellow: at risk for STR, blue: first tertial with RA/RV ratio <1.02, purple: second tertial with RA/RV ratio 1.02-1.32, red: third tertial with RA/RV ratio >1.33. *STR: secondary tricuspid regurgitation.*

**Supplemental Figure 2: Long-term Kaplan-Meier survival analysis of patients with secondary tricuspid regurgitation (STR) with severe STR divided into quartiles**

**
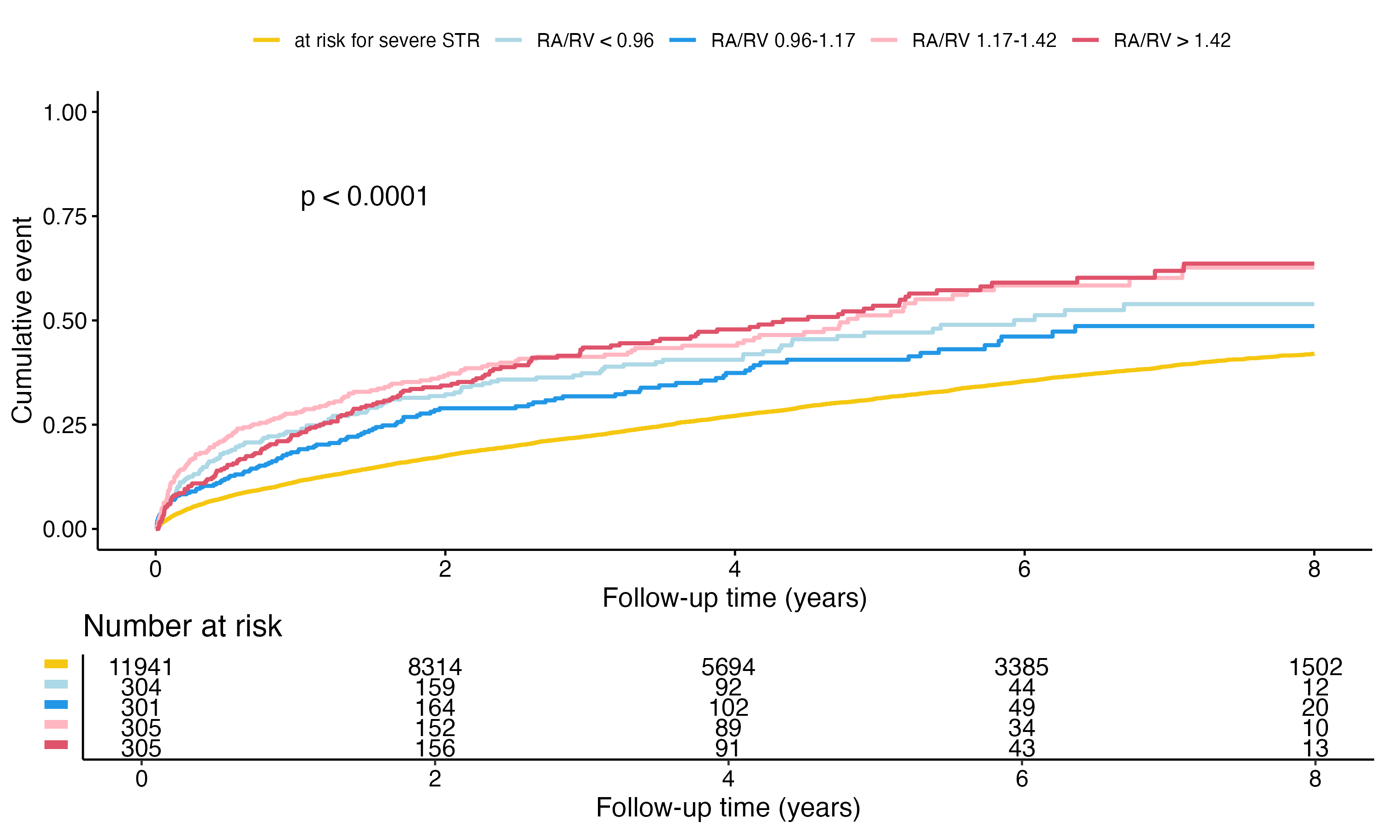
**

Log-rank p>0,001. Yellow: at risk for STR, light blue: first quartile with RA/RV ratio < 0.96, blue: second quartile with RA/RV ratio 0.96-1.17, light red: third quartile with RA/RV ratio 1.17-1.42, red: fourth quartile with RA/RV ratio >1.42. *STR: secondary tricuspid regurgitation.*

**Supplement Figure 3: forest plot of predominant atrial remodeling subgroup analysis**

**
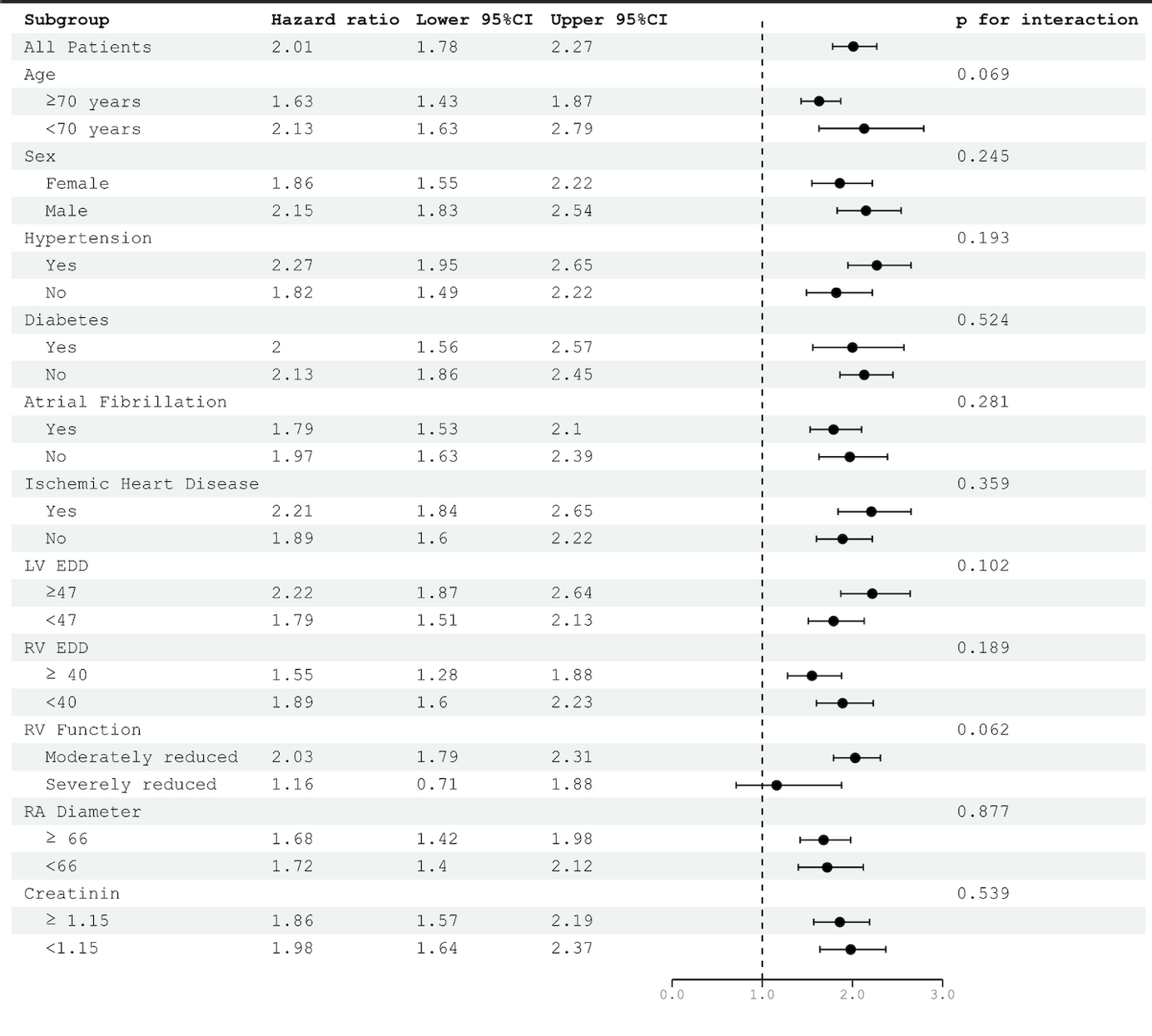
**Forest plot of subgroups of predominant atrial remodeling, showing no significant differences in the subgroup analysis of these patients*. LVEDD: left ventricular end-diastolic diameter, RA: right atrium, RV: right ventricle, RVEDD: right ventricular end-diastolic diameter.*

**Supplement Figure 4: forest plot of predominant ventricular remodeling subgroup analysis**


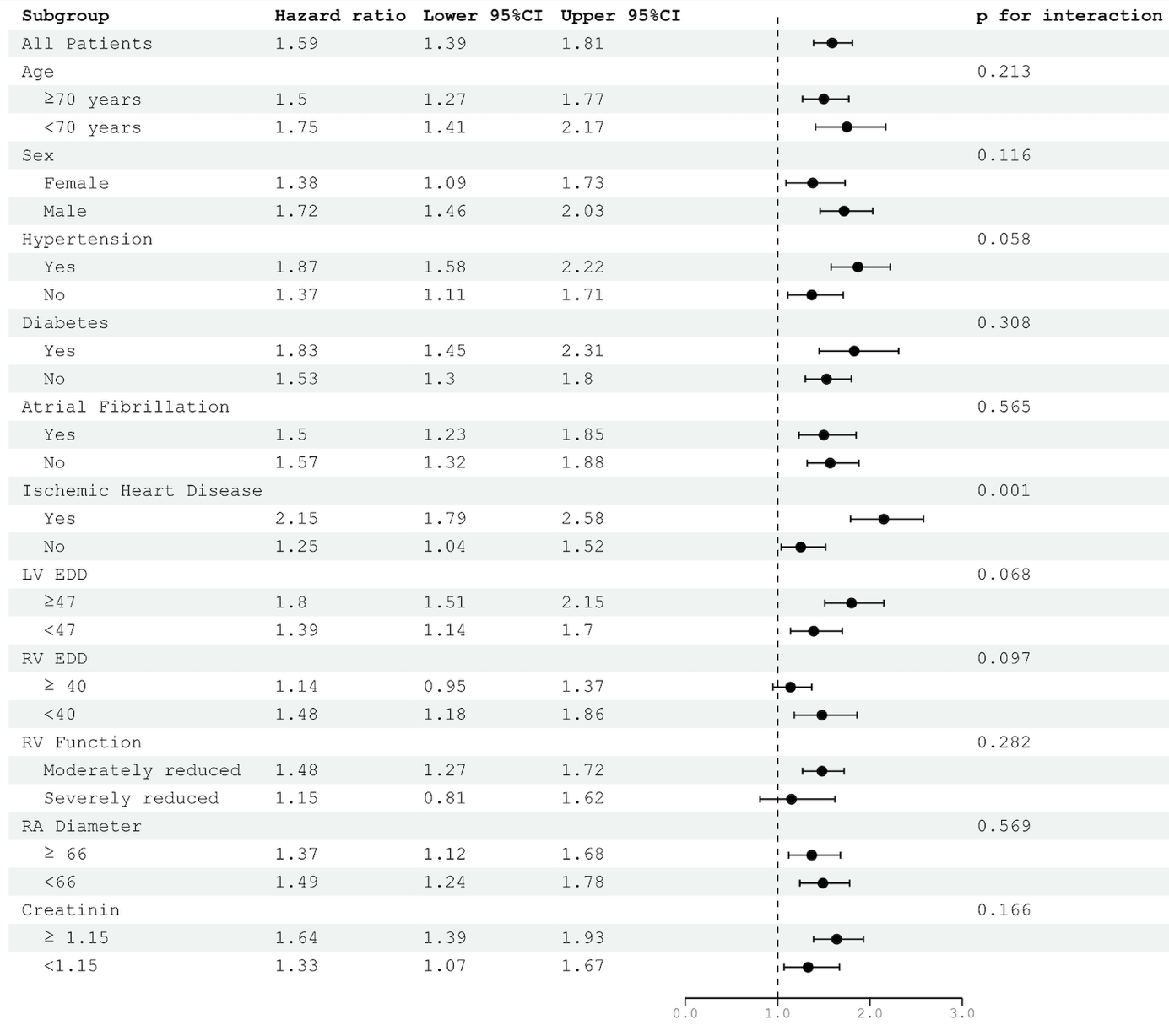
Forest plot of subgroups of predominant ventricular remodeling, showing significant differences in the subgroup analysis of patients with and without ischemic heart disease. *LVEDD: left ventricular end-diastolic diameter, RA: right atrium, RV: right ventricle, RVEDD: right ventricular end-diastolic diameter*
